# Usefulness of voxel-based lesion mapping for predicting motor recovery in subjects with basal ganglia hemorrhage: A preliminary study with 2 case reports

**DOI:** 10.1097/MD.0000000000003838

**Published:** 2016-06-10

**Authors:** Dae Hyun Kim, Sunghyon Kyeong, Yoona Cho, Tae-min Jung, Sung Jun Ahn, Yoon Ghil Park

**Affiliations:** a Department of Physical Medicine and Rehabilitation, Veterans Health Service Medical Center, Seoul; b Severance Biomedical Science Institute, Yonsei University College of Medicine; c Department of Rehabilitation Medicine and Rehabilitation Institute of Neuromuscular Disease, Yonsei University College of Medicine; d Department of Radiology, Yonsei University College of Medicine.

**Keywords:** basal ganglia hemorrhage, cerebral hemorrhage, diffusion tensor imaging, motor-evoked potentials, prognosis, stroke

## Abstract

It is important to estimate motor recovery in the early phase after stroke. Many studies have demonstrated that both diffusion tensor tractography (DTT) and motor-evoked potentials (MEP) are valuable predictors of motor recovery, but these modalities do not directly reflect the status of the injured gray matter. We report on 2 subjects with basal ganglia hemorrhage who showed similar DTT and MEP findings, but had markedly different clinical outcomes. Specifically, Subject 1 showed no improvement in motor function, whereas Subject 2 exhibited substantial improvement 7 weeks after onset. To determine if differences in gray matter might lend insight into these different outcomes, we analyzed gray matter lesions of the 2 subjects using a novel voxel-based lesion mapping method. The lesion of Subject 1 mainly included the putamen, thalamus, and Heschl's gyri, indicating extension of the hemorrhage in the posterior direction. In contrast, the lesion of Subject 2 mainly included the putamen, insula, and pallidum, indicating that the hemorrhage extended anterior laterally. These differential findings suggest that voxel-based gray matter lesion mapping may help to predict differential motor recovery in subjects with basal ganglia hemorrhage with similar DTT and MEP findings.

## 1 Introduction

Motor weakness is one of the most common impairments in patients with basal ganglia hemorrhage (BGH). Most of the motor recovery following stroke occurs within 3 months of onset, and early predictions of long-term outcome of motor function are critically important for rehabilitation planning.^[1–4]^ Therefore, recent studies have attempted to predict motor outcomes in patients with stroke using diffusion tensor tractography (DTT) and/or motor-evoked potential monitoring (MEP).^[2,5]^

It has been well-demonstrated that DTT is a means to noninvasively visualize the course and integrity of major white matter tracts, which positively correlates with motor outcome in subjects with stroke.^[2,5–7]^ Several studies have also reported on the predictive value of MEP for motor outcome in subjects with stroke.^[8,9]^ Jang et al suggested that MEP and DTT have different advantages in predicting motor outcome.^[5]^ However, there is no widely applied analytical method focused on gray matter lesions, and this might be especially useful when DTT and MEP findings are not correlated with motor recovery. The aim of the present study was to compare the differences between 2 patients with BGH with similar DTT and MEP findings, but different motor outcomes, using a novel voxel-based lesion mapping method that is able to reconstruct delimited lesions in gray matter.

## 2 Methods

### 2.1 Subjects

This study retrospectively reviewed 2 cases of BGH. Subject 1 was a 40-year-old male patient with right BGH. Subject 2 was a 50-year-old male patient with left BGH. Both subjects underwent conservative management for BGH and an inpatient rehabilitation program for 3 weeks. Both subjects had sufficient cognitive function to be able to cooperate during the clinical evaluations. Clinical evaluation was performed twice: at the time of transfer into the department of rehabilitation medicine (4 weeks after onset) and on the day of discharge (7 weeks after onset). Magnetic resonance imaging (MRI) and MEP recordings were conducted on the transfer day. The institutional review board, Gangnam Severance Hospital Yonsei University College of Medicine, approved the procedures and protocols of this study.

### 2.2 Clinical evaluation

The Medical Research Council (MRC) and Fugl–Meyer assessment (FMA) were used for evaluation of motor recovery of the affected extremities. The modified Barthel Index (MBI) was obtained to determine the level of independence in activities of daily living. The Functional Ambulation Categories (FAC) assessment was used for determination of walking ability on the basis of the amount of physical support required.^[10]^

### 2.3 Motor-evoked potential recordings

MEP recordings were performed using a MagPro R100 magnetic stimulator (Medtronic Inc., Shoreview, MN) connected to a figure-of-eight coil (diameter = 75 mm). Stimulation was delivered over the motor hotspot of the affected abductor pollicis brevis muscle. The stimulation location was established as the equivalent point of the scalp in the affected hemisphere relative to the unaffected motor hotspot of the abductor pollicis brevis muscle. The absence of an MEP response was confirmed at a stimulation intensity of 100% of maximal stimulation output.

### 2.4 Magnetic resonance image acquisition

All images were acquired with a 3-T clinical whole-body magnetic resonance scanner (GE Signa, Milwaukee, WI) using a 32-channel head coil. A high-resolution 3D T1-weighted image and diffusion tensor image were obtained. The 3D T1-weighted imaging parameters were as follows: TR/TE = 8.29/3.28, thickness = 1 mm, field of view = 240 × 240 mm^2^, matrix = 256 × 256 (reconstructed to 512 × 512), flip angle = 12, reconstructed voxel = 0.430 × 0.430 × 1 mm. Diffusion tensor imaging parameters were as follows: field of view = 240 × 240 mm^2^, matrix 256 × 256, NEX = 1, 32 directions, and *b* = 1000 s/mm^2^; the slice thickness was 2.4 mm for Subject 1 and 4.0 mm for Subject 2.

### 2.5 Diffusion tensor tractography

Head motion effects and image distortion due to eddy currents were corrected using the Oxford Centre for Functional Magnetic Resonance Imaging of the Brain (FMRIB) Software Library (FSL; www.fmrib.ox.ac.uk/fsl). Fiber tracking was performed using the fiber assignment continuous tracking (FACT) algorithm within DTI-studio software version 2.4.01 (http://www.mristudio.org).^[11,12]^ Fiber tracts passing through both regions of interest (anterior mid-pons and low-pons of corticospinal tract on the color map) were designated as the final tracts of interest.^[6]^ Fiber tracking was performed with a fractional anisotropy (FA) threshold of >0.2 and a direction threshold of <60°.^[5]^

### 2.6 Voxel-based lesion mapping

To quantify the full extent of the lesion the volume and area affected by the hemorrhage, we developed a novel voxel-based lesion mapping method. The procedure was as follows: first, borders of each individual's lesion were drawn on the high-resolution T1-weighted image in the native space using MRIcro software (http://www.mricro.com); second, the T1-weighted and lesion images of Subject 2 were flipped to locate the lesion area within the same hemisphere; third, each individual's T1-weighted image and native space lesion image were nonlinearly transformed to the standardized Montreal Neurological Institute (MNI) space using SPM8 (http://www.fil.ion.ucl.ac.uk/spm); lastly, we calculated the percentage of the lesion area that overlapped 116 regions of interest in the automated anatomical labeling (AAL) template.^[13]^ For the last step, we used an in-house Matlab program.

## 3 Results

### 3.1 Clinical evaluation

The MRC, FMA, MBI, and FAC assessments of Subject 1 changed minimally between the transfer day and the discharge day. In contrast, those of Subject 2 were much improved over the same period (Tables 1 and 2).

**Table 1:**
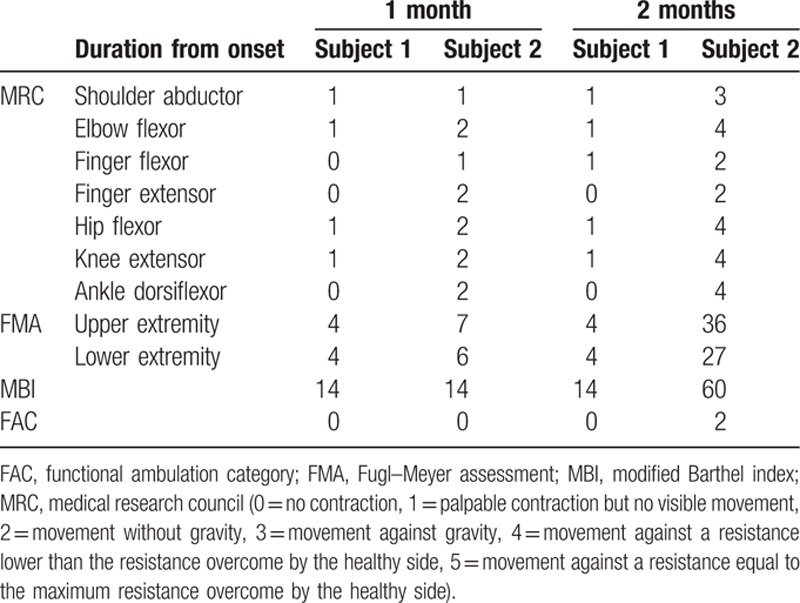
Clinical data of each subject.

**Table 2:**
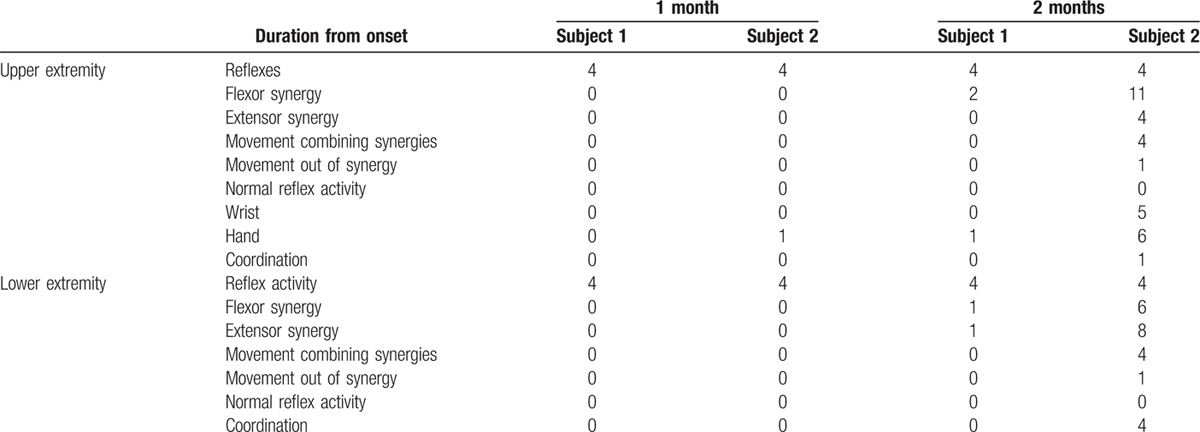
Subscale data of the Fugl–Meyer assessment.

### 3.2 Motor-evoked potentials

No MEPs were evoked from the affected hemisphere. Acceptable MEPs were evoked from the unaffected hemisphere in both subjects.

### 3.3 Diffusion tensor imaging

**Figure 1:**
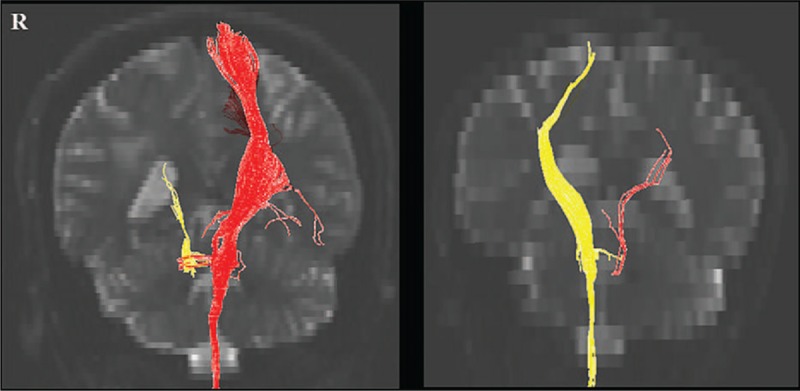
Results of diffusion tensor tractography of the corticospinal tract (left, Subject 1; right, Subject 2). Yellow, right corticospinal tract; red, left corticospinal tract.

The corticospinal tract in the affected hemisphere of each subject was discontinued around the hematoma. The corticospinal tract of unaffected hemisphere was intact in both subjects (Fig. 1).

### 3.4 Voxel-based lesion mapping

**Figure 2:**
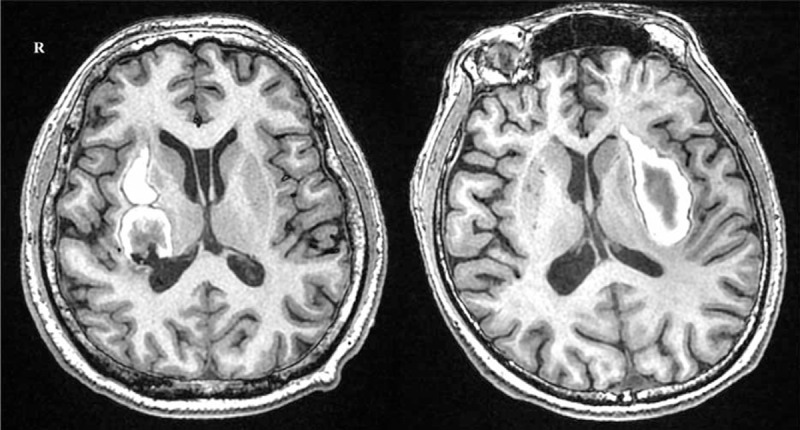
3D T1-weighted images of the primary lesion (left, Subject 1; right, Subject 2).

**Figure 3:**
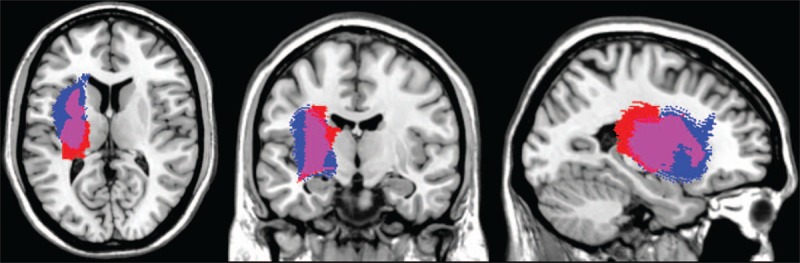
Results for voxel-based lesion mapping. Red, Subject 1; Blue, Subject 2; Pink, region where the lesions overlap in Subjects 1 and 2.

Using our novel method for voxel-based lesion mapping, we determined the volumes of the delimited lesions of Subjects 1 and 2 were 28.3 and 30.6 cc, respectively. The lesion of Subject 1 mainly involved the putamen, thalamus, and Heschl's gyri, overlapping with 45.2%, 18.4%, and 3.75% of the gray matter of these areas, respectively. These findings indicate that BGH extended in the posterior direction (Figs. 2 and 3). The lesion of Subject 2 mainly involved the putamen, insula, and pallidum, overlapping with 38%, 18.7%, and 6.4% of the gray matter of these areas, respectively. These findings indicate that the BGH extended in the anterior-lateral direction (Figs. 2 and 3).

## 4 Discussion

In this study, we introduced a novel voxel-based gray matter lesion mapping method to predict motor outcome at the early stage of BGH. Our results indicated that subjects with similar MEP and DTT abnormalities can have markedly different motor outcome. In this scenario, voxel-based gray matter lesion mapping may be a valuable predictive tool, as it enables precise characterization of the delimited lesion outside of the white matter.

It is becoming increasingly clear that DTT and MEP performed at an early stage of basal ganglia hemorrhage have limitations in predicting motor outcome. Although some controversy exists, previous DTT studies have demonstrated that DTT has a predictive value for motor outcome in subjects with intracerebral hemorrhage.^[2,6,14,15]^ Jang et al demonstrated that early DTT findings of abnormalities in the corticospinal tract may predict motor outcome in the affected extremities, but detailed assessment of the data indicates that FAC scores in subjects with similarly disrupted DTT findings were highly variable 6 months after onset (FAC scores were 0 in 10% of subjects, 3 in 30%, and 4 in 60%).^[6]^ TMS also has been actively researched as a tool for predicting motor outcome,^[16–18]^ but thus far has yielded a rather poor negative predictive value.^[9]^

In this study, we demonstrated that voxel-based lesion mapping allows the visualization of injured areas of BGH, which differed based on the direction of hemorrhage extension in 2 subjects with notably different motor outcomes. The results are similar to previous studies reporting that BGH prognosis varies according to anatomical location.^[19,20]^ The findings of this study and these previous reports indicate that the potential for recovery from motor weakness was better in lateral-type than in posterior-type lesions.^[19,20]^ This may be because the posterior limb of the internal capsule is adjacent to the basal ganglia. As a result, damage to the posterior BGH is likely to directly affect the pyramidal tract, whereas lateral BGH damage might merely compress the pyramidal tract. However, DTT and MEP may not be able to distinguish between direct damage and mere compression of the pyramidal tract in the early stages after stroke.

Voxel-based lesion mapping enables the characterization of the injured gray matter by using the AAL template. Although the integrity of the corticospinal tract can be visualized by DTT, the results are reflective only of the course of major white matter tracts.^[2]^ However, the basal ganglia consists of a set of subcortical gray matter nuclei, such as the caudate nucleus, putamen, globus pallidus, substantia nigra, and subthalamic nuclei,^[20]^ and thus, DTT cannot directly reflect the status of injured nuclei in the basal ganglia. Moreover, hematomas that develop in the basal ganglion areas vary in size and the direction they extend in, and these factors lead to different functional outcomes in BGH. Using voxel-based lesion mapping, we were able to not only determine the extent of hematoma spreading, but also quantify the percentage of injured subcortical nuclei in AAL. Such quantifiable data may be useful in explaining the broad spectrum of clinical manifestations of BGH.

There are a couple of notable limitations to the present study. First, it was a retrospective study. Second, as a preliminary study on the novel voxel-based lesion mapping method, our method was applied to just 2 patients with different hemispheric lesions thus allowing for selection bias. Further studies involving a larger number of cases are required for a more precise, thorough, and reliable characterization of BGH. Third, the slice thickness used in diffusion tensor imaging was not the same for each subject. However, a 4-mm thickness is commonly used, and thickness does not necessarily influence DTT findings.^[2]^

In conclusion, voxel-based lesion mapping may be helpful in predicting motor outcome in patients in the early stage of BGH. It is a particularly useful method for patients who show no recordable MEP and discontinued corticospinal tract in the affected hemisphere, but in whom motor recovery continues to occur within a few months after onset. The quantifiable data of the injured gray matter can be used to stratify patients based on the severity of damage to the gray matter, and to identify the individual functions of specific brain areas in future studies.
